# Characterization and Comparison of the Leukocyte Transcriptomes of Three Cattle Breeds

**DOI:** 10.1371/journal.pone.0030244

**Published:** 2012-01-23

**Authors:** Wen Huang, Asif Nadeem, Bao Zhang, Masroor Babar, Morris Soller, Hasan Khatib

**Affiliations:** 1 Department of Dairy Science, University of Wisconsin, Madison, Wisconsin, United States of America; 2 Institute of Biochemistry and Biotechnology, University of Veterinary and Animal Sciences, Lahore, Pakistan; 3 College of Animal Science and Technology, Northwest A&F University, Yangling, Shaanxi, China; 4 Department of Genetics, The Hebrew University of Jerusalem, Jerusalem, Israel; 5 Department of Animal Sciences, University of Wisconsin, Madison, Wisconsin, United States of America; Emory University School of Medicine, United States of America

## Abstract

In this study, mRNA-Seq was used to characterize and compare the leukocyte transcriptomes from two taurine breeds (Holstein and Jersey), and one indicine breed (Cholistani). At the genomic level, we identified breed-specific base changes in protein coding regions. Among 7,793,425 coding bases, only 165 differed between Holstein and Jersey, and 3,383 (0.04%) differed between Holstein and Cholistani, 817 (25%) of which resulted in amino acid changes in 627 genes. At the transcriptional level, we assembled transcripts and estimated their abundances including those from more than 3,000 unannotated intergeneic regions. Differential gene expression analysis showed a high similarity between Holstein and Jersey, and a much greater difference between the taurine breeds and the indicine breed. We identified gene ontology pathways that were systematically altered, including the electron transport chain and immune response pathways that may contribute to different levels of heat tolerance and disease resistance in taurine and indicine breeds. At the post-transcriptional level, sequencing mRNA allowed us to identify a number of genes undergoing differential alternative splicing among different breeds. This study provided a high-resolution survey of the variation between bovine transcriptomes at different levels and may provide important biological insights into the phenotypic differentiation among cattle breeds.

## Introduction

There are two major types of modern cattle breeds which diverged 610,000–850,000 years ago [1]. The taurine (*Bos taurus*) breeds, originating primarily from Europe and the northern parts of Asia, are adapted to temperate climates. The indicine (*Bos indicus* or *Bos taurus indicus*) breeds are of South Asian origin and evolved in warm tropical climates [2]. There exists substantial phenotypic difference between taurine and indicine cattle. In particular, they differ remarkably in their resistance to thermal stress, parasites, and diseases [3], [4]. In this study, the transcriptomes of three cattle breeds were investigated. Holstein and Jersey are two common taurine dairy breeds predominantly found in Europe and North America, and Chlolistani is an indicine dual-purpose (dairy and meat) breed found in the Cholistan desert region in Pakistan where annual rainfall averages at 180 cm and mean temperature is 28.3°C [5]. The Cholistani breed is renowned for its remarkable tolerance to heat and resistance to ticks and diseases [5].

The recent sequencing and identification of abundant DNA variants for the bovine genome have significantly facilitated the analysis of genetic divergence between cattle breeds [6], [7]. Population genetic analysis has revealed a clear separation of animals into distinct genetic clusters consistent with their breed identity and relatedness, implying large between-breed genetic variation [7]. However, little is known regarding the expression of genes in cattle and the variation between breeds.

The importance of understanding transcriptomic variation is obvious as the role of gene expression in shaping phenotypes is well documented. In particular, the transcriptomic variation among cattle breeds may provide mechanistic knowledge on their differentiation on phenotypes including appearance, physiological, behavioral, and production traits. There is accumulating evidence that variation in gene expression, presumably controlled by genomic variations within regulatory elements, contributes to phenotypic variation. For example, variation in expression of certain genes including the ornithine aminotransferase was associated with nicotine resistance in fruit flies [8]. Furthermore, in an evolutionary context, differences in gene expression are important in determining phenotypic differentiation of closely related species such as primates whose similarity in DNA sequences do not appear to fully explain the distinct phenotypes [9]. Importantly, while the expression of most genes stays unchanged evolutionarily, there are a significant number of genes whose expression levels are under natural selection, suggesting their involvement in adaptation and evolution [9], [10]. In addition, regulation of gene expression is far more complicated than steady-state messenger RNA (mRNA) levels. Additional modes of regulation exist at different stages in the life cycle of mRNA, including splicing that generates alternative isoforms and mRNA sequestration that prevents its translation. Variation in these stages of regulation may also be under natural selection and contribute to phenotypic variation. For example, two recent studies in primates have reported lineage-specific alternative splicing in the brain and liver transcriptomes [11], [12].

In this study, we used mRNA-Seq to characterize and compare the leukocyte transcriptomes of Holstein, Jersey, and Cholistani with respect to variations in sequence, expression, and splicing. These variations may provide partial explanations for differential phenotypes between cattle breeds, particularly between *taurus* and *indicus* cattle.

## Materials and Methods

### Ethics Statement

This study was approved by the Animal Care and Use Protocol committee of the Research Animal Resources Center at the University of Wisconsin-Madison.

### RNA preparation

Tail vein blood was collected from 40 Holstein cows at the University of Wisconsin Dairy Cattle Center, 7 Jersey cows at the University of Wisconsin Arlington Agricultural Research Station, and 45 Cholistani cows at GujaitPeer Farm, Bahawalpur, Punjab, Pakistan. Immediately following blood sampling, equal volumes of whole blood were pooled before white blood cells were isolated by centrifugation at 3000 g for 15 min. The white blood cells were washed twice with red blood cell lysis buffer, followed by RNA extraction using the Qiagen RNAEasy Mini Kit (Qiagen, Valencia, CA). For the Cholistani breed, RNA was transported in RNAStable (Biomatrica, San Diego, CA), a storage matrix mimicking an anhydrobiotic environment, to preserve its integrity. We quality checked all RNA samples using Agilent Bioanalyzer 2100 (Agilent, Santa Clara CA) before generation of sequencing libraries.

### Library generation and sequencing

Polyadenylated RNA was selected using magnetic oligo dT beads and subjected to Illumina's mRNA-Seq library generation. Briefly, mRNA was fragmented, reverse transcribed, adapted with sequencing primers, size selected (∼300 bp), and PCR enriched. The resulting library for each breed was sequenced on one lane of an Illumina Genome Analyzer IIx. Approximately 20 million fragments were sequenced by 75 bp from both ends.

### Mapping reads to the reference genome

We used Tophat (v1.0.14) to align mRNA-Seq reads to the reference genome, which used Bowtie (v0.12.7) as the aligner [13] and a segment mapping algorithm to discover splice junctions [14]. To maximize sensitivity of splice junction discovery, we first discovered novel splice junctions in each sample independently. The splice junctions were then combined and supplied to Tophat together with known splice junctions from the Ensembl annotation (release 60). We allowed a maximum of two mismatches in each of the three 25-bp segments and discarded reads that aligned to more than 20 genomic locations. For identification of breed-specific bases within breeds, we focused on uniquely mapped reads and required each base be covered by at least 10 reads. To identify breed-specific bases, we filtered out putative polymorphic bases by the following criteria: 1) at least 10% of the reads supported the minor allele; 2) the alleles are supported by at least one read on both strands with quality value ≥20; and 3) the two most frequent alleles accounted for at least 90% of the reads.

### Assembly of transcripts and estimation of abundance

The resulting alignments were used to reconstruct transcript models and estimate their abundance in the transcriptomes by Cufflinks (v0.9.3) [15]. Cufflinks assembles transcript models by finding the minimum number of transcripts supporting the overlapping alignments at disjoined genomic loci. In addition to transcript assembly, Cufflinks also estimates gene expression (fragments per kilobase exon per million mapped fragments, or FPKM) by finding parameters that maximize the likelihood function of observing fragment coverage given the transcript models. Abundances of transcripts were upper-quartile normalized [16] and corrected for sequence bias [17], which was done internally in Cufflinks [18].

### Identification of differentially-expressed genes, isoforms, and GO enrichment analysis

We tested for differential expression of genes and isoforms using Cuffdiff, a companion tool of Cufflinks for testing differential expression and regulation [15]. Genes or isoforms with FDR <0.05 were considered significant. To test for enrichment of GO terms in differentially-expressed genes, we used the GOSeq (v1.2.0) package in R [19]. GOSeq explicitly takes into account gene selection bias due to difference in gene lengths and thus numbers of overlapping sequencing reads [19]. P values reported by GOSeq were corrected using the Benjamini-Hochberg procedure to obtain FDR [20].

### Validation of differentially-expressed genes

For validation of differential expression by real time RT-PCR, we used the same RNA samples for sequencing from Holstein and Cholistani and synthesized cDNA using the iScript cDNA synthesis kit (Bio-rad, Hercules, CA). Real time PCR was performed in triplets according to the manufacturer's instruction using the iQ SYBR Green (Bio-rad, Hercules, CA) on a Bio-rad iCycler (Bio-rad) and analyzed by the 2^−ΔΔCt^ method using *GAPDH* as the reference control [21]. Primer sequences are listed in Table S1. Primers are designed in Beacon Designer with considerations of amplicon length, annealing temperature and secondary structures, among others (PREMIER Biosoft International, Palo Alto, CA). All primers were designed such that the amplicons span at least one intron in the primary transcripts.

## Results

### Sequencing of the bovine transcriptomes

To characterize the transcriptomes of cattle breeds, we collected blood samples from Holsteins and Jerseys (taurine breeds), and Cholistanis (indicine breed). These three breeds represented two distinct groups of cattle. While the taurine cattle are adapted to and primarily found in temperate environments, the indicine cattle are adapted to tropical climates.

Blood samples from multiple animals were pooled to obtain an “averaged” transcriptome in each breed. RNA was extracted from leukocytes isolated from blood pools. Following Illumina Genome Analyzer IIx's standard procedure, approximately 21 million paired-end fragments were sequenced for each of the three breeds (Table 1). We aligned sequence reads to the bovine reference genome (btau4.0) using Tophat, an RNA-Seq aligner that is capable of discovering splice junctions de novo [14]. The Ensembl annotation was supplied to Tophat to ensure the sensitivity of aligning spliced fragments to known junctions. Approximately 70% of the sequenced fragments were aligned successfully by allowing no more than two mismatches in 25 bp segments and restricting alignments to at most 20 genomic locations (Table 1). Among the aligned fragments, over 90% were mapped to unique genomic regions (Table 1).

**Table 1:** Summary of sequencing read alignments.

Sample	Holstein	Jersey	Cholistani
Total sequenced fragments	21,078,477	21,358,931	20,940,063
Fragments mapped to the nuclear genome	13,565,995	14,384,823	16,403,447
(percent mapped)	(64.4%)	(67.3%)	(78.3%)
Uniquely mapped fragments	12,373,228	13,104,182	15,203,561
(percent uniquely mapped)	(91.2%)	(91.1%)	(92.7%)

### Transcript sequence variation among cattle breeds

**Figure 1:**
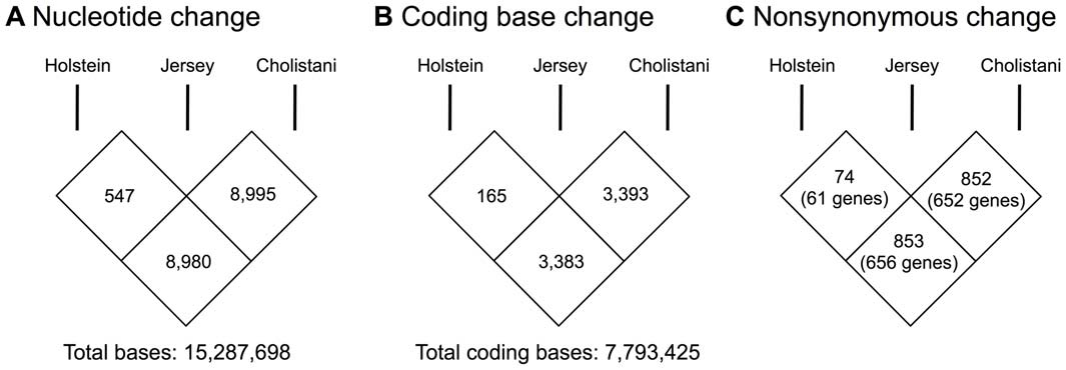
Nucleotide and protein diversity among cattle breeds. Each number in a diamond-shaped box represents the number of differences between two breeds indicated by the flanking vertical bars. Differences in nucleotides (A), coding bases (B), and non-synonymous bases (C) are shown.

The sequencing nature of mRNA-Seq allowed us to interrogate sequence variations of expressed mRNAs. Focusing on reliably-called bases (≥10 read coverage) and those that did not vary within each breed, we found 15,287,689 nucleotides whose identities could be determined in all three breeds. Among these bases, 547 (0.004%) differed between Holstein and Jersey; 8,980 (0.059%) between Holstein and Cholistani; and 8,995 (0.059%) between Jersey and Cholistani (Figure 1A). In addition, 7,793,425 of these bases fell within the protein-coding regions of 8,870 genes. Among these coding nucleotides, 165 differed between Holstein and Jersey, whereas 3,383 and 3,393 differed between Holstein and Cholistani, and between Jersey and Cholistani, respectively (Figure 1B). Although most of the coding base changes were synonymous, there were 74, 853, and 852 nonsynonymous substitutions in the pair-wise comparisons between Holstein and Jersey, Holstein and Cholistani, and Jersey and Cholistani, respectively (Figure 1C). Taken together, these results indicate very high nucleotide and protein sequence similarities between the three cattle breeds, although the difference between taurine and indicine breeds was over ten-fold greater than that between the two taurine breeds.

To gain biological insights to the genomic differences between breeds, we asked whether there was an enrichment of certain biological pathways in the genes that contained non-synonymous base changes between breeds. Because the sequences of Holstein and Jersey were extremely similar, we chose Holstein as a representative for taurine cattle and compared it against Cholistani. Only genes with 50% coding region covered by at least 10 fragments were included in the analysis (n = 5,624). Although this did not reveal GO terms reaching the FDR cutoff (0.05), the “innate immunity response” GO term appeared to be highly enriched for genes with non-synonymous substitutions between Holstein and Cholistani; seven out of the 19 genes in this GO term contained non-synonymous base changes (nominal p = 0.0007).

### Variation of overall gene expression among cattle breeds

While transcript sequences reflect ultimately the genetic makeup of different breeds and amino acid changing bases may affect functions of proteins, the abundances of transcripts or genes can also express phenotypic variation. To estimate transcript abundance, we first used overlapping and spliced alignments, and paired-end information to construct transcript models *ab initio*. We used Cufflinks to assemble transcripts in each of the three breeds [15]. We recovered 11,063, 10,320, and 8,291 transcripts in Holstein, Jersey, and Cholistani, respectively, that were compatible with existing genome annotation. Restricting the analysis to transcripts whose exon-exon junctions were supported by at least two sequenced fragments, we discovered a combined total of 11,050 unannotated spliced isoforms of known genes. Additionally, Cufflinks assembled 4,362 transcripts in 3,426 intergenic regions (at least 1,000 bp from known genes on either strand), representing 3,242,073 unannotated bases. We combined newly-identified spliced isoforms and intergenic transcripts with the known annotated genes to obtain a non-redundant set of transcript models containing 25,968 genes and 42,837 transcripts, for which isoform-specific and overall gene expression were estimated using Cufflinks.

**Figure 2:**
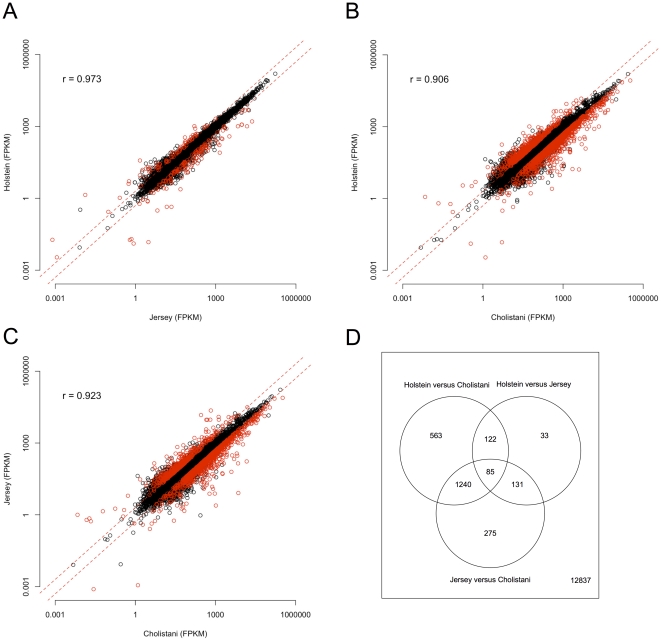
Comparison of overall gene expression between cattle breeds. Gene expression from each of the three breeds was plotted against each other: Holstein versus Jersey (A); Holstein versus Cholistani (B); and Jersey versus Cholistani (C). Also shown in each scatter plot were two dashed lines indicating 2-fold change. Red circles indicated genes differentially expressed significantly by at least two fold. In (D), a Venn diagram was shown for overlap between genes that showed significant differential expression in each of the three pair-wise comparisons.

We compared gene expression of the leukocyte transcriptomes of Holstein, Jersey, and Cholistani. Among the 15,286 genes that could be detected in any of the three breeds, 13,409, 13,787, and 13,666 were detected in Holstein, Jersey, and Cholistani, respectively (Figure S1). Out of these, 375, 560, and 983 genes were breed-specific in Holstein, Jersey, and Cholistani, respectively. Breed-specific transcripts were found to be lowly expressed in general (Figure S2), therefore the specificity of many may in fact be a result of mRNA-Seq not being able to detect the transcripts. As expected, transcriptomes of the two taurine breeds exhibited very high similarity (Pearson's correlation r = 0.973, Figure 2A), whereas the similarities between Cholistani and Holstein (r = 0.906, Figure 2B) and between Cholistani and Jersey (r = 0.923, Figure 2C) were substantially lower.

**Figure 3:**
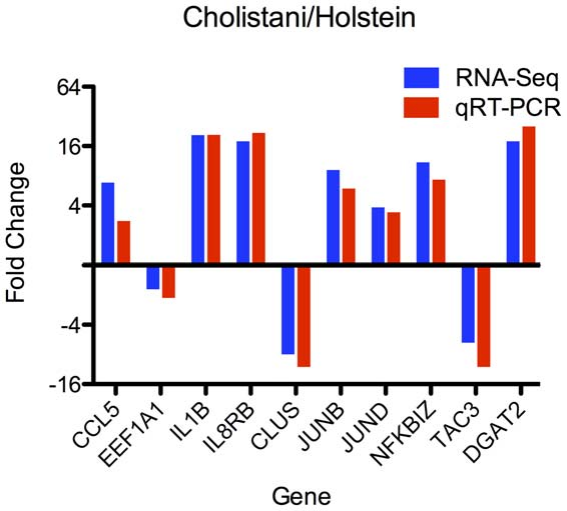
Fold changes measured by mRNA-Seq and real time RT-PCR.

We then asked how many genes were differentially expressed between breeds. Controlling false discovery rate (FDR) at 0.05, we found 371 genes that showed at least a 2-fold difference between Holstein and Jersey. On the other hand, 2,010 and 1,731 genes were found to be significantly different (≥2 fold) between Holstein and Cholistani, and between Jersey and Cholistani, respectively. Importantly, among the genes that differed between Holstein and Cholistani, or between Jersey and Cholistani, the majority (n = 1240) showed no difference between the two taurine breeds but consistent difference between the indicine breed and either taurine breed (Figure 2D). To test whether our sequencing and analysis were valid and reliable, we selected 10 differentially-expressed genes and measured their expression in the same RNA samples of Holstein and Cholistani by real-time RT-PCR. All ten genes showed uniformly consistent results in real-time RT-PCR and sequencing (Figure 3).

Overrepresentation of 1,723 annotated genes that showed differential overall expression between Holstein and Cholistani in 3,288 GO biological processes and 1,769 GO molecular function categories was then tested. At FDR = 0.05, a total of 12 GO terms showed significant overrepresentation in the genes differentially expressed between Holstein and Cholistani (Table 2). Interestingly, we found significant regulation of GO biological processes including “electron transport”, “translation” “immune response”, and “inflammatory response” (Table 2).

**Table 2:** Significant (FDR <0.05) GO enrichment in differentially-expressed genes between Holstein and Cholistani.

GO terms ID	Description	FDR (q value)
*Molecular Function*		
GO:0003735	structural constituent of ribosome	<0.0001
GO:0005515	protein binding	<0.0001
GO:0004129	cytochrome-c oxidase activity	0.0001
GO:0008009	chemokine activity	0.0072
GO:0008137	NADH dehydrogenase (ubiquinone) activity	0.0112
GO:0003954	NADH dehydrogenase activity	0.0255
*Biological Process*		
GO:0022900	electron transport chain	<0.0001
GO:0006412	translation	<0.0001
GO:0006955	immune response	0.0002
GO:0006954	inflammatory response	0.0002
GO:0006935	chemotaxis	0.0018
GO:0045941	positive regulation of transcription	0.0467

### Variation of alternative splicing among cattle breeds

**Figure 4:**
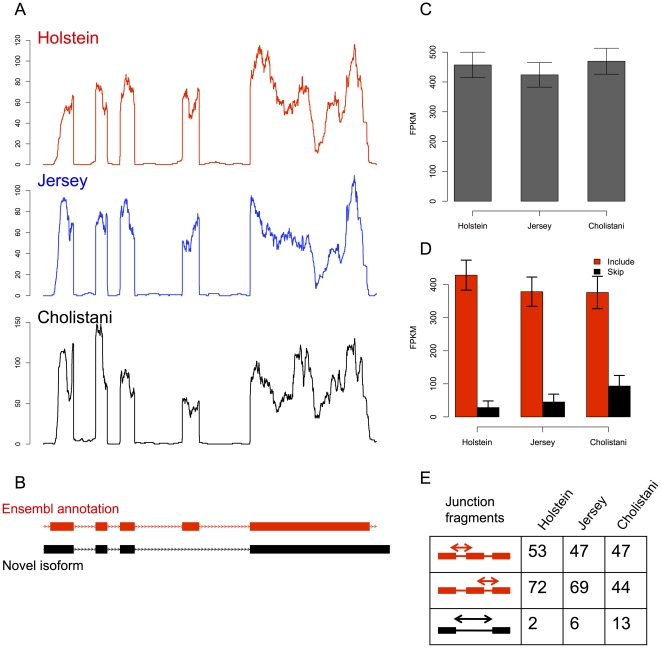
Example (*TMED4*) of differential alternative splicing between breeds. Fragment coverage (A) for each of the three breeds was plotted along the transcript models (B). Note the relative abundance of exon 4. Overall gene expression was largely unchanged (C) whereas relative abundance showed a significant difference between breeds (D). Numbers of junction fragments (E) also supported the differential alternative splicing.

Difference in protein sequences can be not only a consequence of non-synonymous nucleotide substitutions, but also a consequence of differences in protein isoforms generated by alternative splicing events. In addition, transcript isoforms may contain different regulatory elements that alter mRNA stability and translation efficiency. Based on existing and newly-identified transcript models, we tested differential splicing regulation using Cuffdiff [15]. Cuffdiff takes isoform expression estimates from Cufflinks, measures the distribution of relative expression between different isoforms originated from the same transcription using the metric Shannon-Jensen divergence, and assigns statistical significance for comparison between samples [15]. Because 5′ ends of transcripts critically depended on sequencing depth and gene expression, we conservatively focused on 2,134 genes with a single transcription start site (TSS) inferred from the RNA-Seq data and that expressed more than one isoform. Isoforms of these genes were classified into a total 3,002 alternative splicing events that fell into six categories (Table 3). Because we conservatively investigated genes with a single TSS, alternative first exon was not interrogated. We found 355, 711, and 660 genes undergoing significant differential alternative splicing (FDR <0.05) for the comparison between Holstein and Jersey, between Holstein and Cholistani, and between Jersey and Cholistani, respectively. For example, we discovered an unannotated isoform of the bovine *TMED4* (transmembrane emp24 protein transport domain containing 4) gene (ENSBTAG00000010612), which skipped the fourth exon of its primary transcript. The overall expression of this gene was similar in three breeds. However, relative abundance of the two isoforms differed significantly between the taurine and indicine breeds, suggesting a breed-specific regulation of alternative splicing (Figure 4).

**Table 3:** Alternative splicing events detected in genes and significant differential alternative splicing between Holstein and Cholistani.

	Total	Exon skipping^1^	Alternative 5′ splice site	Alternative 3′ splice site	Intron retention	Mutually exclusive	Alternative last exon
Events	3,002	1,498	444	884	837	59	117
Genes	2,134	740	258	480	398	43	81
Significant genes(Holstein vs. Cholistani)	711	312	100	190	164	17	24

1 Alternative splicing events were classified as in Wang et al. 2008 [27].

Finally, we also tested for enrichment of biological pathways among the genes that showed differential alternative splicing between Holstein and Cholistani. Interestingly, although not reaching the controlled FDR level, two related GO terms “structural constituent of ribosome” and “translation” showed nominal significance (p = 0.0007 and 0.0014 respectively). Importantly, although the same GO terms were significantly enriched for differential overall gene expression (Table 2), the overlap between genes that showed differential overall expression and differential alternative splicing was minimal. For example, among the 26 “structural constituent of ribosome” and 28 “translation” genes, only six also showed differential overall expression between Holstein and Cholistani. This suggested that the two pathways were systematically altered at both the transcriptional and splicing level.

## Discussion

In this study, we performed high throughput mRNA sequencing of leukocyte transcriptomes of three cattle breeds. Following alignment of sequencing reads, we first evaluated the nucleotide and protein sequence divergence, which showed little difference between the two taurine breeds (Holstein and Jersey), but considerably larger difference between taurine can indicine (Cholistani) breeds. We then reconstructed transcript models and estimated their abundances, and identified genes that showed differential expression or alternative splicing. Among the differentially-expressed genes between Holstein (taurine) and Cholistani (indicine), we found enrichment for genes involved in several biological pathways including electron transport, translation, and immune responses. In addition, we found differential alternative splicing for a number of genes, suggesting that the breed-specific regulation of gene expression occurs at multiple levels including transcription as well as splicing. These results provide important biological insights into the phenotypic differentiation

We first characterized sequence variation among cattle breeds. A small proportion of the monomorphic nucleotides were found to be different among breeds. Even though the difference was substantially larger than that between Holstein and Jersey, there were fewer than 9,000 (out of 15,287,698; 0.059%) base changes between Holstein and Cholistani. For comparison, we also identified putative single nucleotide polymorphisms (SNPs) within each breed. Among 22,100,344 surveyed bases in Holstein, 32,547 (0.147%) were reliably called SNPs; and 39,370 out of 19,967,581 (0.197%) bases were polymorphic within Cholistani. The number of polymorphic bases could not be directly interpreted as within-breed diversity. Nevertheless, the fact that there were considerably more SNPs within breeds than monomorphic base differences between breeds suggests that the between-breed nucleotide divergence is indeed small and unlikely to fully account for the phenotypic differences between breeds.

Among the amino acid-changing bases found in this study, one in the Cholistani introduced a premature stop codon that resulted in a truncation of the five amino acids of the p21 protein activated kinase 1 interacting protein 1 (PAK1IP1, ENSBTAG00000018674) gene. No difference was detected for expression of this gene among the three breeds. Human PAK1IP1 is widely expressed in multiple tissues and has been shown to interact with p21-activated kinase 1 (PAK1) and negatively regulate various activities of the PAK1 pathway [22], including cell survival, mitosis, and cytoskeletal organization [23]. However, the consequences of this mutation in the Cholistani breed and its relevance to the phenotypic differences between the taurine and indicine breeds remain to be determined. Another interesting finding was the enrichment of the “innate immune response” pathway genes in the list of genes that contained non-synonymous base changes between Holstein and Cholistani. Unlike variation in gene expression that may be a result of transient response to environmental factors, changes in nucleotide/protein sequences are heritable and may represent genetic divergence among breeds due to evolutionary forces such as natural and artificial selections.

Because sequence variation at the nucleotide and protein levels did not seem to explain the diverse phenotypes among breeds, particularly between taurine and indicine breeds, we also looked for variation at the transcriptional level. Indeed, there existed substantial difference among breeds (Figure 2). However, it is difficult to attribute variation in gene expression to breed identities as opposed to random variation between animals. One has to obtain a large enough sample and statistically test the hypothesis that variation among breeds is larger than individual variation within each breed. This is prohibitive for high throughput sequencing. Instead of sequencing animals individually from each breed, we pooled samples to obtain an ‘averaged’ transcriptome that was representative of the breed. Although we lost information on individual variability of gene expression, the estimate of mean gene expression in each breed was accurate because of the relatively large numbers of animals pooled. The averaging of transcriptomes by pooling blood samples reduces variation in gene expression estimates due to variability between individuals. By comparing means of gene expression instead of gene expression of single animals, it also allows us to make biological inference.

Cattle breeds differ in many aspects including local environment, nutritional input, and management, many of which also influence gene expression. These environmental variables may partially confound breed identities, presenting a difficulty in attributing variation in gene expression to breed effect alone. We believe it is more sensible to define a breed as a combination of its ancestry, genetic makeup, the local environment it adapts to, and other breed-specific variables. In other words, the variation observed in the transcriptomes of different cattle breeds reflects the overall effects of all breed-defining genetic and non-genetic factors.

The biological process GO term “electron transport chain” was significantly enriched for differentially-expressed genes between Holstein and Cholistani (Table 2). Low metabolic rate is a major player in thermotolerance for *Bos indicus* [4]. Given the key role of the electron transport chain in energy metabolism, the change in expression of this pathway may be associated with the remarkable difference in thermotolerance between Holstein and Cholistani. We also observed an enrichment of genes showing differential expression between Holstein and Cholistani involved in the immune response and inflammatory response of animals (Table 2). The Cholistani breed is particularly well known for its resistance to ticks and other diseases [5]. A recent transcriptomic study in peripheral leukocytes from tick-infested *Bos taurus* and *Bos indicus* showed significant difference for genes in various biological processes including metabolic and immune response processes between the two cattle breeds [24]. Consistent with this result, our study further supports the importance of expression of the immune system genes in different susceptibilities to parasites among breeds.

In addition to overall gene expression, mRNA-Seq also allowed us to characterize and compare alternative splicing in the transcriptomes. Indeed, we identified over 3,000 alternative splicing events in 2,134 genes analyzed and found a large number of differential alternative splicing among breeds (Table 3). Although not reaching controlled FDR level, two GO pathways (“structural constituent of ribosome” and “translation”) enriched for differential overall expression also showed considerable enrichment in genes showing differential alternative splicing. This result suggested that differential regulation of gene expression among breeds was at multiple levels including transcriptional and post-transcriptional. One example of the differential alternative splicing was the *TMED4* gene that encodes a transmembrane protein associated with the endoplasmic reticulum membrane. The variant skipping the fourth exon, which disrupts the coding region of this gene, was found to be significantly more frequent in Cholistani than in its taurine counterparts (Figure 4). Interestingly, previous studies have indicated an important role of this protein in heat shock response. In one study, depletion of expression of *TMED4* resulted in a decrease in apoptosis in response to heat stress [25]. Another study showed that viability of lymphocytes in response to heat shock was significantly higher in *indicus* breed than in *taurus* breed [26]. The preferential switching of a functional isoform to a non-functional isoform of *TMED4* in Cholistani, thus reducing heat shock-induced apoptosis, may in part explain the breed's remarkable tolerance to heat.

In conclusion, our study is among the first to use high throughput sequencing for characterization of bovine transcriptomes. Not only have we discovered genes that show breed-specific expression pattern, we have also found that regulation of gene expression exists at multiple levels. These results may provide important insights into genes that are associated with adaptation and specialization of cattle breeds.

## Supporting Information

Figure S1 **Venn diagram showing overlap and uniqueness of genes expressed in Holstein, Jersey, and Cholistani.**
(TIFF)

Figure S2 **Histograms of gene expression for all genes and breed-specific genes.**
(TIFF)

Table S1 **Primer sequences for real time RT-PCR.**
(DOC)
