# Supplementary figures and images for: Characterization and Comparison of the Leukocyte Transcriptomes of Three Cattle Breeds
Source: PLoS One. 2012 Jan 23;7(1):e30244. doi: 10.1371/journal.pone.0030244 (PMC3264571; doi:10.1371/journal.pone.0030244)

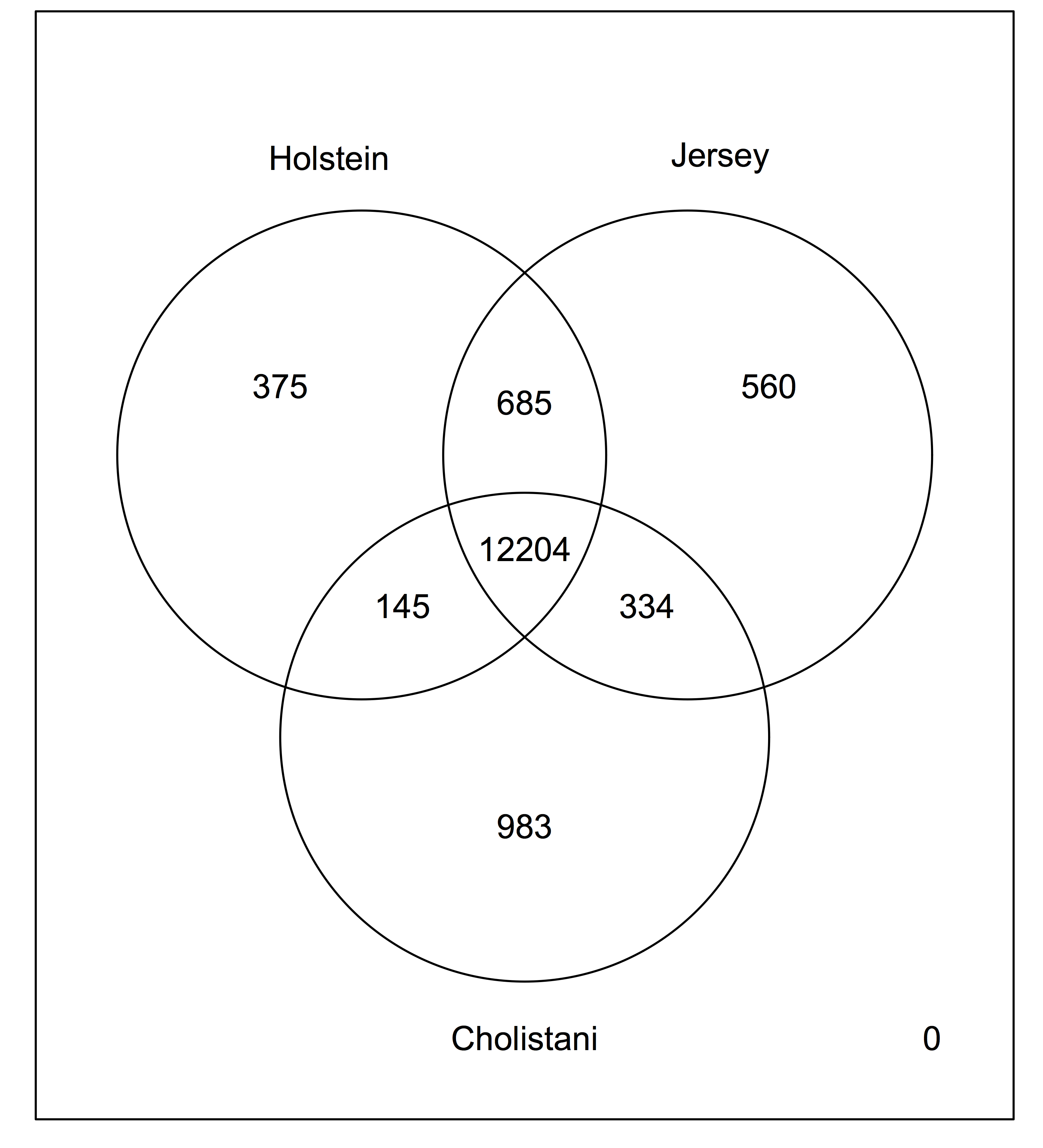

Supplement: Figure S1 — Venn diagram showing overlap and uniqueness of genes expressed in Holstein, Jersey, and Cholistani. (TIFF) [file pone.0030244.s001.tif]

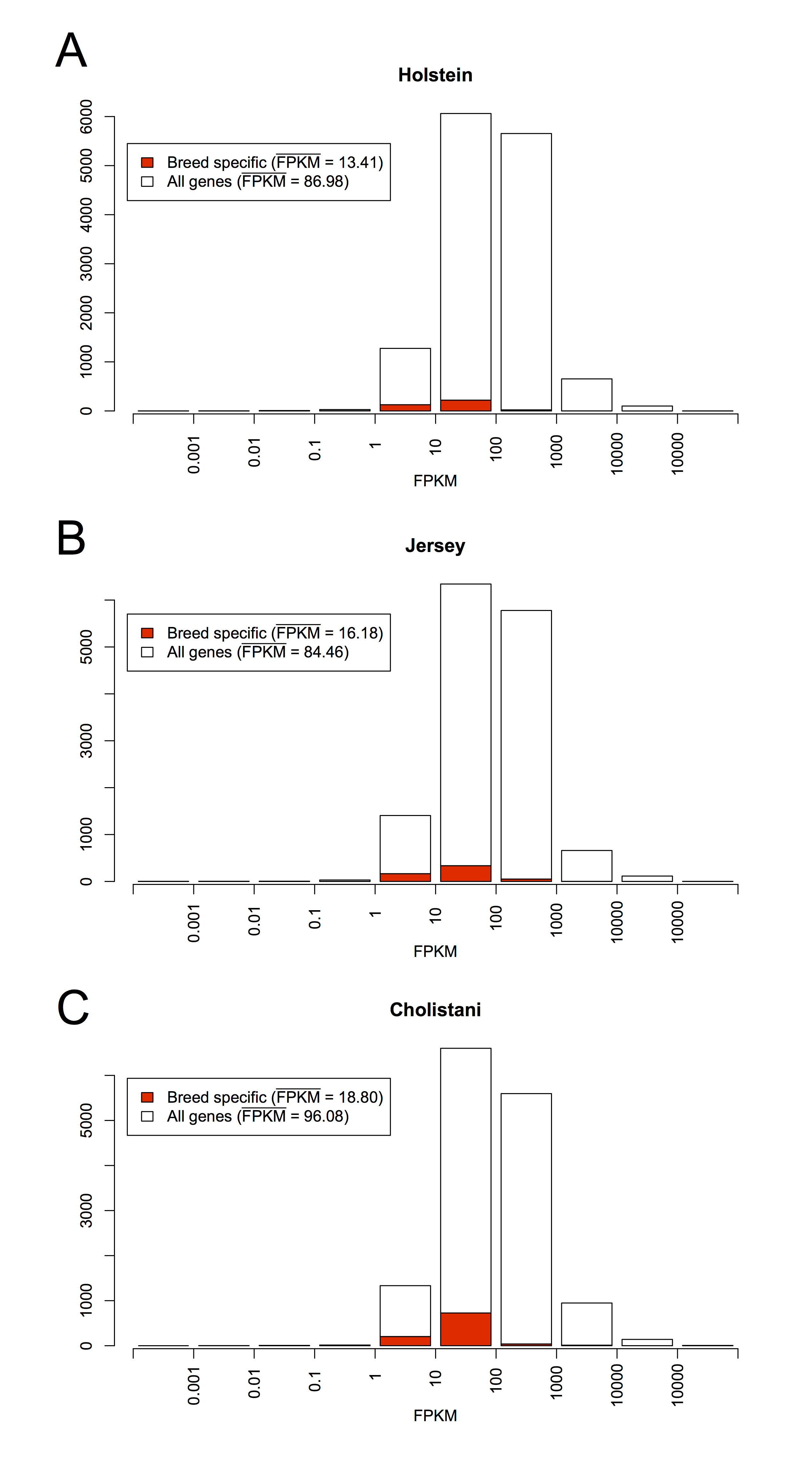

Supplement: Figure S2 — Histograms of gene expression for all genes and breed-specific genes. (TIFF) [file pone.0030244.s002.tif]
