# Supplementary material for: Characterization and Comparison of the Leukocyte Transcriptomes of Three Cattle Breeds
Source: PLoS One. 2012 Jan 23;7(1):e30244. doi: 10.1371/journal.pone.0030244 (PMC3264571; doi:10.1371/journal.pone.0030244)
Supplement: Table S1 — Primer sequences for real time RT-PCR. (DOC) [file pone.0030244.s003.doc]

Table S1. Primer sequences for real time RT-PCR

| Gene | Strand | Sequence (5’ – 3’) |
| --- | --- | --- |
| CCL5 | Forward | ATGGCAGCAGTTGTCTTT |
|  | Reverse | TACTCTCGCACCCACTTC |
| EEF1A1 | Forward | AGGACACAGAGACTTCAT |
|  | Reverse | CAAATTCACCAACACCAG |
| IL1B | Forward | AGGAAATGAACCGAGAAG |
|  | Reverse | ACACAAGACAGGTATAGATT |
| IL8RB | Forward | GAAGTTCTGATTTGTAGCATTTG |
|  | Reverse | AACACCACCATCCTTGAA |
| CLUS | Forward | CACGATGGAATTCACAGA |
|  | Reverse | GATCTCCTGGCACTTCTC |
| JUNB | Forward | CCAGTCCTTCCACCTCAA |
|  | Reverse | TCCTCCCCTCCCTGTTAA |
| JUND | Forward | TTTGACTTTGGAAGAGAGAAC |
|  | Reverse | AACCAACACAGGAGAACA |
| NFKBIZ | Forward | GAACTTGGAGAACGAACA |
|  | Reverse | TCTCTGCTGAATGGACTT |
| TAC3 | Forward | TCACAGCAAGAAGGACTC |
|  | Reverse | TCACAGCAAGAAGGACTC |
| DGAT2 | Forward | TCACAGTGGGTCCGAAAC |
|  | Reverse | ATGTAGTTCCTGCTGGTCA |
